# Gag Mutations Strongly Contribute to HIV-1 Resistance to Protease Inhibitors in Highly Drug-Experienced Patients besides Compensating for Fitness Loss

**DOI:** 10.1371/journal.ppat.1000345

**Published:** 2009-03-20

**Authors:** Elisabeth Dam, Romina Quercia, Bärbel Glass, Diane Descamps, Odile Launay, Xavier Duval, Hans-Georg Kräusslich, Allan J. Hance, François Clavel, ANRS 109 Study Group

**Affiliations:** 1 Inserm U552, Paris, France; 2 BioalliancePharma, Paris, France; 3 Viralliance Inc., Paris, France; 4 Institut Universitaire d'Hématologie, Hôpital Saint-Louis, Paris, France; 5 Department of Virology, Universitätsklinikum Heidelberg, Heidelberg, Germany; 6 Laboratoire de Virologie, Hôpital Bichat-Claude Bernard, Paris, France; 7 Faculté de Médecine Paris Descartes and CIC de vaccinologie Cochin Pasteur, Paris, France; 8 Centre d'Investigation Clinique, Hôpital Bichat-Claude Bernard, Paris, France; University of Geneva, Switzerland

## Abstract

Human immunodeficiency virus type 1 (HIV-1) resistance to protease inhibitors (PI) results from mutations in the viral protease (PR) that reduce PI binding but also decrease viral replicative capacity (RC). Additional mutations compensating for the RC loss subsequently accumulate within PR and in Gag substrate cleavage sites. We examined the respective contribution of mutations in PR and Gag to PI resistance and RC and their interdependence using a panel of HIV-1 molecular clones carrying different sequences from six patients who had failed multiple lines of treatment. Mutations in Gag strongly and directly contributed to PI resistance besides compensating for fitness loss. This effect was essentially carried by the C-terminal region of Gag (containing NC-SP2-p6) with little or no contribution from MA, CA, and SP1. The effect of Gag on resistance depended on the presence of cleavage site mutations A431V or I437V in NC-SP2-p6 and correlated with processing of the NC/SP2 cleavage site. By contrast, reverting the A431V or I437V mutation in these highly evolved sequences had little effect on RC. Mutations in the NC-SP2-p6 region of Gag can be dually selected as compensatory and as direct PI resistance mutations, with cleavage at the NC-SP2 site behaving as a rate-limiting step in PI resistance. Further compensatory mutations render viral RC independent of the A431V or I437V mutations while their effect on resistance persists.

## Author Summary

Protease inhibitors are among the most active antiviral drugs used in the treatment of Human immunodeficiency virus type 1 (HIV-1) infection. The efficacy of these compounds, however, can be threatened by the emergence of viral resistance, the result of the gradual accumulation of specific mutations in the viral protease. HIV-1 resistance to protease inhibitors often results in impaired protease function and in the loss of the replicative capacity of the virus, an effect that can be partially corrected by selection of compensatory mutations in one of the natural substrates of the protease, the Gag protein. In this study, we have found that Gag mutations not only correct viral replicative capacity but also play a major and direct role in resistance. We observed that this effect is essentially mediated by mutations in the C-terminal region of Gag, and that it correlates with the extent of cleavage downstream of the Gag nucleocapsid protein. Our results establish that mutations in Gag constitute a second and important pathway of HIV-1 resistance to protease inhibitors in patients failing antiretroviral treatment.

## Introduction

The Human Immunodeficiency virus type 1 (HIV-1) protease (PR) is a key enzyme in viral replication and a major target for therapeutic intervention. Protease inhibitors (PI) are the backbone of some of the most active combinations of antiretroviral drugs used in the treatment of HIV-infected patients. The long-term efficacy of these compounds, however, is threatened by the emergence of viral resistance and subsequent spread of resistant virus. HIV-1 resistance to PIs is promoted by gradual accumulation of amino-acid substitutions in PR, resulting in altered PI binding [1]–[5]. Resistance-promoting changes in PR generally also decrease viral replicative capacity (RC) due to decreased processing of the natural substrate. Accordingly, additional mutations accumulate in PR over time that mainly compensate for these losses in RC, but may also contribute to resistance directly [1]–[3],[6]. The biological effect of PI resistance mutations thus has to be viewed as the product of their effect on enzyme inhibition (resistance) and on enzyme activity (RC).

**Figure 1:**
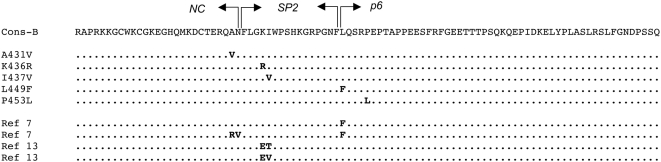
Principal mutations in the NC-SP2-P6 region of HIV-1 Gag selected in viruses exposed to protease inhibitors *in vivo* or *in vitro*. The upper part of the figure lists cleavage site mutations most often observed in viruses exposed to protease inhibitors *in vivo*. The cleavage site mutations shown in the lower part of the figure were selected by *in vitro* passage of laboratory strains of HIV-1 in the presence of protease inhibitors as described in Doyon et al. [7] and in Nijhuis et al. [13].

Besides mutations directly affecting PR, several mutations in the Gag polyprotein, the main substrate of PR, have been found to play a significant role in the evolution of PI resistance [7]–[13]. These mutations were generally classified as compensatory mutations that restore activity of the mutated PR for its natural substrate [7]–[9],[12],[14],[15]. Particular attention has been turned to mutations in the region surrounding the NC-SP2-P6 cleavage sites at the C-terminus of Gag. Figure 1 gives an overdrive of these mutations and of their position in the NC-SP2-p6 region of HIV-1 Gag. The most frequently observed cleavage site mutations in this region are substitution A431V, located at position P2 of the NC-SP2 cleavage site, mutation L449F at position P1' of the SP2-P6 cleavage site, and mutations K436R and I437V, situated immediately downstream of the NC-SP2 site. Interestingly, some of these mutations appear to depend upon the presence of specific mutations in PR: Mutation A431V is mostly observed in PI-resistant viruses carrying mutations V82A and/or M46I in PR [16]. Mutation L449F is frequently seen in viruses with mutation I84V in PR [16]. Neither of these two substitutions are seen in wild-type, protease inhibitor-naïve viruses. Substitution P453L is a polymorphism found in some inhibitor-naïve viruses. It is, however, seen with significantly higher frequency in resistant viruses carrying the I84V mutation in PR [16] and also specifically seen in resistant viruses carrying the I50V PR mutation, in association with L449F [12].

In this context, two main questions remain to be answered. First, although some studies have identified Gag polymorphisms outside of cleavage sites that appeared to be important for the replicative capacity of viruses with PR mutations [15]–[20], the extent to which changes in the matrix (MA), capsid (CA), nucleocapsid (NC) proteins and in adjoining cleavage sites participate in evolution of viruses under PI pressure *in vivo* has not been fully evaluated. Second, the potential impact of Gag cleavage site mutations on viral resistance, in addition to their well established compensatory effect on viruses carrying PI resistance mutations, remains to be established. Nijhuis et al. [13] recently reported the emergence of viruses carrying mutations K436E and I437V (Figure 1) within the SP2 linker peptide of Gag following *in vitro* selection for HIV-1 resistance to an experimental PI. These mutations in Gag preceded the detection of mutations in PR, and decreased HIV susceptibility to several PIs. It is currently not clear, however, whether mutations in Gag have a selective impact on the level of resistance under PI pressure *in vivo*, and whether this impact could be independent of the effect of these mutations on viral RC.

In this study, we have constructed recombinant viruses carrying different Gag-Pol segments from the plasma of HIV-1 infected subjects in whom viral resistance had evolved to high levels following prolonged antiretroviral treatment failure. Recombinant viruses were tested phenotypically for resistance and replicative capacity. In most cases, the loss of RC resulting from resistance mutations in PR was partially compensated by the NC-SP2-P6 region of Gag while addition of the MA and CA domains had no effect. The presence of patient-derived NC-SP2-P6 resulted in strong increases in the IC50 of most PIs, establishing the important role of this region in determining resistance. Selective reversion of mutations A431V or I437V in a subset of these recombinant viruses produced a strong reduction in resistance, but only a minor effect on replication capacity. The effects on viral resistance correlated with processing at the NC-SP2 cleavage site, revealing the importance of this cleavage event for HIV-1 resistance to PIs. Our results suggest a second pathway of resistance in patients failing a PI-containing regimen, establish a direct effect of Gag cleavage site mutations on antiviral resistance beyond their previously described compensatory role, and identify NC-SP2 cleavage as a limiting step in resistance development *in vivo*.

## Results

### Construction of recombinants carrying patient-derived Gag-Pol sequences

**Figure 2:**
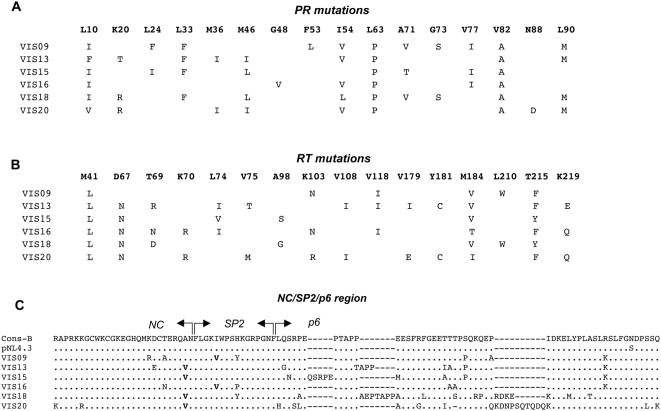
Genotypes of the viruses used in the study. (A) Protease resistance genotypes, showing mutations at positions identified as critical for resistance to protease inhibitors according to the IAS-USA resistance mutations table, as shown at http://www.iasusa.org/resistance_mutations/mutations_figures.pdf. (B) Reverse transcriptase resistance genotypes. (C). Aminoacid polymorphisms in the NC-SP2-p6 region of Gag, relative to the sequence of NL4-3.

Gag-Pol HIV-1 sequences from 6 patients were studied. The PR and RT sequences of these viruses contained multiple resistance mutations, as defined according to the IAS-USA table, and are shown on Figure 2A (PR) and Figure 2B (RT). The mean number of mutations in PR was 8.8 (range: 6–11). All 6 viruses also carried mutations in the NC-SP2-P6 cleavage region of Gag (Figure 2C). Mutation A431V, at the P2' position of the NC-SP2 cleavage site was found in 4 viruses. Mutation I437V, immediately downstream of that cleavage site, was found in 2 viruses, and mutation L449H, at the P1 position of the SP2-p6 cleavage site was found in one virus, in association with the A431V mutation. Interestingly, mutations A431V and I437V were found to be mutually exclusive in these 6 viruses, which was also the case in the 20 other patients of the ANRS 109 study (data not shown).

**Figure 3:**
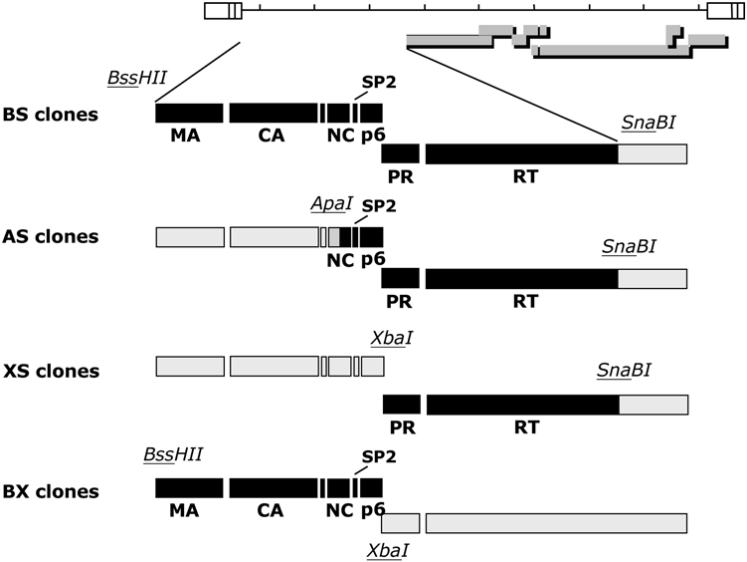
Construction of recombinant clones carrying HIV-1 Gag-Pol sequences from treated patients. Schematic representation of the different segments of Gag-Pol used to create the clones used in the study, as described in Materials and Methods. Black rectangles represent sequences from patient virus; grey rectangles represent sequences from pNL4-3. The restriction enzymes and restriction sites used for the constructions are indicated. MA, matrix protein; CA, capsid protein; NC, nucleocapsid protein; SP2, spacer peptide 2; PR, protease; RT, reverse transcriptase.

For each of these 6 viruses, 4 different Gag-Pol recombinant clones were constructed (Figure 3). Clones BS contained the whole of Gag, PR and RT from patient virus, down to the junction between RT and RNAse H. Clones AS carried patient-derived C-terminal half of NC, SP2 spacer peptide and p6, in association with PR and RT from the same patient. Clones XS had only PR and RT sequences from patient virus in association with NL4-3 Gag. Finally, clones BX carried the whole Gag from patient viruses, in association with NL4-3 PR and RT. It is noteworthy that in the latter, the transframe region of the Gag-Pol polyprotein was patient-derived, while the last 12 amino acids at the C-terminus of the Gag polyprotein were from NL4-3. For each virus, the AS, XS and BX clones were derived from a single BS clone.

### Effect of different segments of Gag on PI resistance

All 4 clones from each of the 6 patients were tested phenotypically for resistance to PIs. The results of these analyses, representing mean±SD of at least 3 independent experiments, are shown on Table 1, where resistance is expressed as a fold-change in IC50 relative to NL4-3. Six distinct PIs were tested: indinavir (IDV), nelfinavir (NFV), amprenavir (APV), saquinavir (SQV), lopinavir (LPV) and atazanavir (ATV). Viruses carrying patient-derived PR, RT and complete Gag sequences (BS clones) were strongly resistant to most PIs tested, in accordance with clinical failure of the respective patients. In contrast, resistance was found markedly lower in viruses carrying only patient-derived PR and RT in absence of any patient-derived Gag sequences (XS clones). This finding emphasizes the critical importance of HIV-1 Gag in viral resistance to PIs. When a shorter segment of Gag encompassing the NC-SP2-P6 region was added to the same highly mutated PR and RT sequences (AS clones), resistance levels were comparable to those measured with clones carrying the full Gag coding sequence (BS clones), strongly suggesting that most of the resistance impact of Gag is restricted to the NC-SP2-p6 region. Therefore, the MA and CA proteins, together with their flanking cleavage sites, do not appear to play a significant role in PI resistance selection *in vivo*, at least for this group of heavily pre-treated patients. Interestingly, all BX clones, which carried patient-derived Gag but wild-type PR and RT, exhibited increased IC50 levels relative to NL4.3, an effect that ranged from 1.6-fold to 5.6-fold, suggesting that even in the presence of wild-type PR, changes in Gag occurring during the course of HIV evolution under selective pressure by PIs *in vivo* exert a clear effect on HIV-1 suseptibility to these compounds.

**Table 1:** Resistance to protease inhibitors and replicative capacity of Gag-recombinant proviral clones.

		IDV*	NFV*	APV*	SQV*	LPV*	ATV*	RC**
**VIS09**	*BS*	43±2.2	31.9±5.4	2.8±0.4	38.8±8.2	98.2±27.1	80.0±36.1	54.4±5.2
	*AS*	57.3±15.2	40.5±10.0	3.5±0.5	57.5±11.3	85.6±22.5	95.5±46.3	**29.3±9.1**
	*XS*	**17.5±6.5**	**16.0±6.0**	**1.2±0.4**	24.1±12.6	**42.9±22.8**	26.8±9.6	**3.7±1.7**
	*BX*	3.7±1.3	2.7±1.3	2.5±0.2	2.5±0.5	5.3±3.1	4.6±1.2	219.6±37.2
**VIS13**	*BS*	173.5±39.6	127.4±47.2	52.9±26.8	20.8±4.7	>221	49.4±13.7	117.9±30.3
	*AS*	>237	173.7±54.7	76.4±24.6	28.9±3.9	>221	72.7±17.1	131.6±25.4
	*XS*	**40.3±12.6**	**43.2±16.5**	**12.5±4.5**	**8.8±6.9**	98±26.7	**8.3±3.4**	**2.2±1.1**
	*BX*	2.3±0.7	2.2±1.1	1.6±0.1	2.4±0.2	3.7±0.3	2.6±0.7	62.2±12.3
**VIS15**	*BS*	33.7±6.1	13.5±5.8	15.4±2.1	1.7±0.3	33.4±15.1	23.0±3.1	61.9±15.1
	*AS*	20.9±4.2	10.2±3.3	11.0±2.5	1.2±0.2	24.6±9.6	**16.2±3.6**	50.4±11.2
	*XS*	**2.5±0.7**	**1.4±0.9**	**1.6±0.6**	**0.2±0.1**	**3.6±1.2**	**2.2±0.8**	**3.4±1.2**
	*BX*	3.8±0.4	3.2±0.7	2.9±0.1	2.1±1.0	5.6±1.8	4.9±2.3	87.8±21.6
**VIS16**	*BS*	35.2±19.6	19.8±10.3	1.3±0.4	59.2±6.1	33.3±8.6	34.8±7.9	31.9±6.0
	*AS*	46.3±12.2	21.4±7.7	1.5±0.4	>66	56.2±22.1	51.1±5.5	22.5±8.5
	*XS*	**9.9±3.5**	**6.6±2.9**	**0.5±0.03**	**21.4±2.7**	16.9±6.4	**15.6±0.9**	14.3±4.1
	*BX*	3.0±0.5	2.6±1.0	1.9±0.2	2.7±0.5	3.7±1.2	3.2±1.6	232.4±39.4
**VIS18**	*BS*	23.2±6.3	19.3±6.9	20.9±2.0	6.5±1.3	31.2±13.6	73.5±13.1	45.3±9.1
	*AS*	30±8.4	28.2±15.4	24.1±1.7	8.3±2.4	32.8±11.6	65.2±22.5	53.5±12.3
	*XS*	**5.4±1.4**	6.9±3.7	**5.9±1.0**	**1.6±0.4**	**7.6±1.6**	**17.1±0.9**	**3.8±1.6**
	*BX*	3.0±0.4	2.0±0.5	1.8±0.9	1.4±0.5	2.3±0.4	1.8±0.4	36±4.3
**VIS20**	*BS*	97.1±29.6	55.9±31.3	3.8±0.7	16.7±3.8	153.3±64.3	78.3±29.3	103.9±8.5
	*AS*	114.1±15.4	65.4±17.1	5.3±1.3	24.5±3.2	187.3±55.2	184.1±50.0	45.7±18.9
	*XS*	**28.7±12.8**	**8.6±0.8**	**0.9±0.2**	**5.6±1.2**	**40.1±8.8**	25.6±2.8	**6.1±1.9**
	*BX*	2.5±0.6	2.9±1.0	2.2±0.4	2.3±0.4	3.8±1.0	3.5±1.2	162.6±48.5

Numbers are the mean of at least three independent experiments±SD.

Bold numbers indicate that the difference between AS or XS values and BS values was found statistically significant using a one-way ANOVA followed by Bonferroni's multiple comparison post-test.

*: Resistance is expressed as a fold-change in IC50 relative to pNL4-3, as described in Materials and Methods.

**: Replicative capacity (RC) is expressed as a % of pNL4-3 RC, as described in Materials and Methods.

IDV, indinavir; NFV, nelfinavir; APV, amprenavir; SQV, saquinavir; LPV, lopinavir; ATV, atazanavir.

### Effect of different regions of Gag on replicative capacity

As expected, the presence of patient-derived Gag sequences had a strong positive impact on the RC of the multiply resistant viral clones. The RC of XS clones, which lacked patient-derived Gag, was markedly lower than that of BS clones, carrying the whole Gag sequence from patient plasma: the mean RC of XS clones was 5.6% of NL4-3, and the mean change in RC for XS clones relative to cognate BS clones was a 19.5-fold decrease. The greatest such effect was seen with the VIS13 virus (53.6-fold decrease) and the lowest with VIS16 (2.2-fold decrease). Similar to what was seen for resistance, the RC of most BS clones did not differ from that of cognate AS clones, again emphasizing the absence of a significant impact of MA and CA on resistance-associated loss of RC. It is noteworthy that substantial variation in RC was seen among the BS clones from different patients, with the lowest (VIS16) at 31.9±6.0% and the highest (VIS13) at 117.9±30.3%. Similarly, marked variation in RC was observed among BX clones, which only carried patient-derived Gag in absence of mutated PR and RT. For 4 of the clones, the RC of BX clones was higher than that of the cognate BS clone, suggesting that resistance mutations in PR and/or RT continued to exert some deleterious effect on RC in spite of compensatory mutations in Gag. For 2 viruses, however, the RC of the BX clone was lower than that of the BS clone (VIS13, VIS18). In these cases, the RC of the BX clone was also lower than that of the AS clone, whereas the RC of BS and AS clones were similar. Taken together, these findings are consistent with the possibility that these viruses carried mutations in the NC-SP2-p6 region able to facilitate replication of viruses with resistance mutations in PR and RT, but whose presence is deleterious for replication of viruses carrying wild-type PR and RT sequences.

Overall, the increase in RC resulting from the presence of patient-derived Gag sequences in clones carrying highly mutated PR and RT sequences was almost strictly parallel to the increase in resistance observed with the same clones. In general, however, the effect measured on RC was stronger than the resistance effect. Thus, for example, the mean change in IDV resistance for XS clones relative to BS clones was a 5.2-fold decrease, compared to the 19.5-fold loss in RC.

### Individual role of NC-SP2 cleavage site mutations A431V and I437V in the ability of patient-derived Gag sequences to enhance RC and resistance

**Figure 4:**
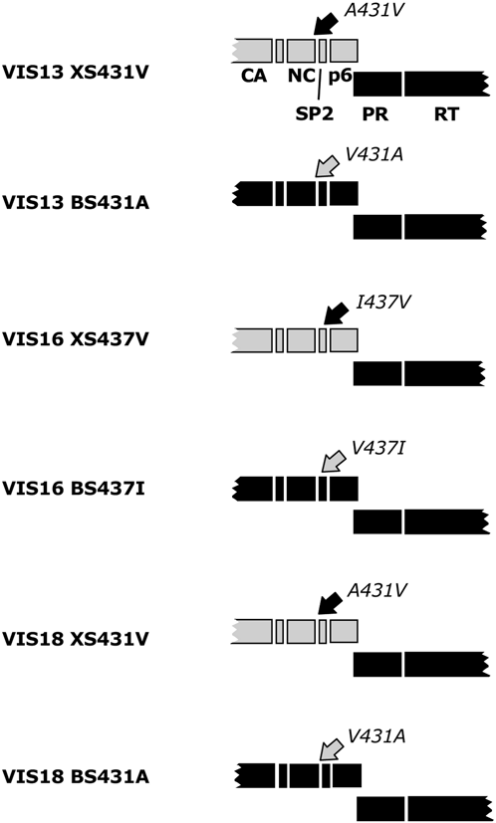
Modification of cleavage site sequences by site-directed mutagenesis. Schematic representation of the mutations reverted or introduced in Gag cleavage sites by site directed-mutagenesis, as described in Materials and Methods. Black rectangles represent sequences from patient virus; grey rectangles represent sequences from pNL4-3. Black arrows indicate introduction of a cleavage site Gag mutation as found in patient-derived resistant virus into NL4-3-derived sequences. Grey arrows indicate reversion of a cleavage site Gag mutation from patient-derived Gag sequences back to wild-type NL4-3 codon.

Having established that the determinants in Gag able to exert an enhancing effect on HIV-1 RC and resistance to PIs are essentially restricted to the NC-SP2-p6 region, we sought to evaluate the precise role of NC-SP2 mutations A431V and I437V in this phenomenon. We therefore selected XS clones from 3 patients and introduced the A431V (seen in VIS13 and VIS18 viruses) or the I437V (seen in VIS16) mutations into the NL4-3-derived Gag sequences, yielding the VIS13XS431V, the VIS16XS437V and the VIS18XS431V mutants (Figure 4). In parallel, these NC-SP2 cleavage site mutations were reverted back to wild-type in the patient-derived Gag sequences of the corresponding BS clones, yielding the VIS13BS431A, VIS16BS437I and VIS18BS431A mutants.

**Figure 5:**
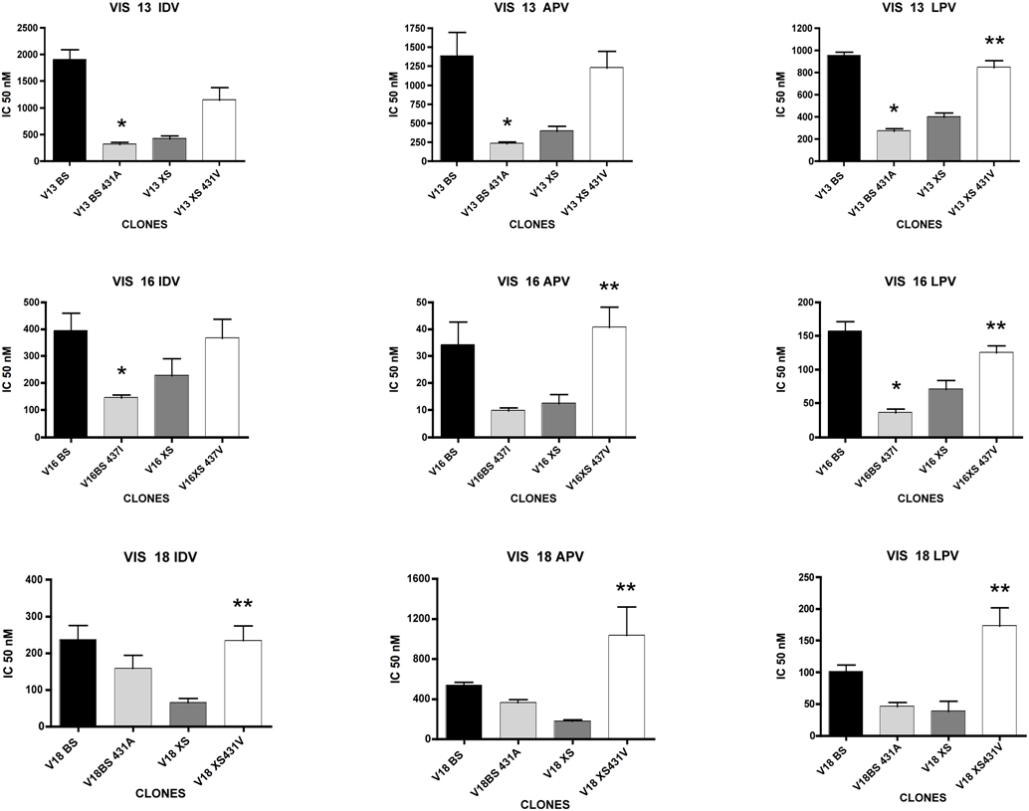
Resistance phenotype of viral clones carrying mutations or reversions in Gag cleavage sites. Each panel shows the IC50 of the indicated protease inhibitor for a set of clones whose construction was based on the same patient-derived HIV-1 sequences. Three sets of clones, from three different patient-derived sequences (VIS13, VIS16, and VIS18) were tested for resistance to indinavir (IDV), amprenavir (APV), and lopinavir (LPV). Black bars, BS clones carrying patient-derived Gag, PR, and RT; light grey bars, clones in which mutation A431V (for VIS13 and VIS 18) or I437V (for VIS16) was reverted back to wild-type in patient-derived Gag in BS clones by site-directed mutagenesis; dark grey bars, XS clones carrying NL4-3 Gag in the presence of patient-derived PR and RT; white bars, clones in which mutation A431V (for VIS13 and VIS 18) or I437V (for VIS16) were inserted in NL4-3-derived Gag in XS clones by site-directed mutagenesis. Single asterisks indicate that the IC50 difference between the indicated clone and the corresponding BS clone is statistically significant. Double asterisks indicate that the IC50 difference between the indicated clone and the corresponding XS clone is statistically significant.

As seen in Figure 5, the reversion of mutations A431V or I437V in BS clones from patients VIS13 and VIS16 resulted in a strong decrease in the IC50 of the three PIs tested, corresponding to a loss of resistance. In these two viral backgrounds, the decrease in IC50 resulting from reversion of the NC-SP2 cleavage site mutations was equivalent to that seen when replacing the whole Gag region from patient virus with that of NL4-3 (i.e., in the XS clones). Coherent with this observation, when mutations A431V and I437V were introduced in the XS clones, a strong increase in IC50 was seen, back to levels close to those seen with the BS clones. This observation strongly suggests that in highly resistant viruses, the resistance impact of the NC-SP2-p6 region is essentially carried by NC-SP2 cleavage site mutations. A slightly different and less clear picture was seen with virus VIS18, where the IC50 effect of reversion of A431V was relatively modest, in particular with IDV, and where this effect did not seem to be quite as pronounced as that seen with the XS clone. Nonetheless, introduction of A431V in VIS18XS produced a strong and significant increase in IC50 to all 3 tested drugs, to levels comparable to those seen with VIS18BS.

**Figure 6:**
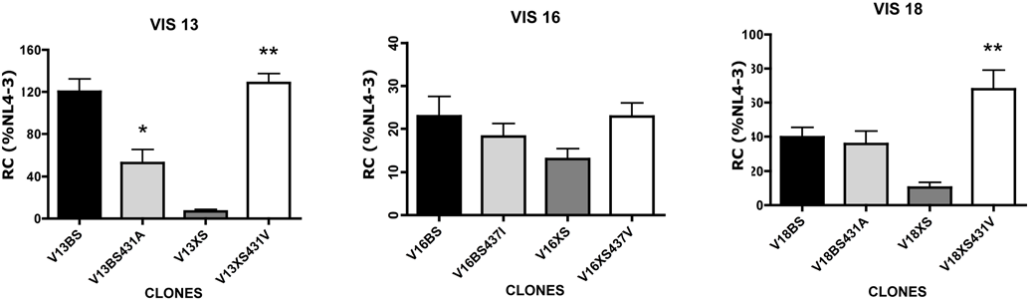
Replicative capacity of viral clones carrying mutations or reversions in Gag cleavage sites. Each panel shows the RC (in % relative to NL4-3 RC) of a set of clones whose construction was based on the same patient-derived HIV-1 sequences. The sets of clones and the color codes are identical to those presented in Figure 4 and described in the legend of Figure 4. Single asterisks indicate that the RC difference between the indicated clone and the corresponding BS clone is statistically significant. Double asterisks indicate that the RC difference between the indicated clone and the corresponding XS clone is statistically significant.

We next examined the effect of these mutations on RC (Figure 6). Reversion of NC-SP2 cleavage site mutations resulted in a moderate or no reduction in RC, contrasting with the much more dramatic effect of these changes on the resistance phenotype. Furthermore, the effect on RC was far less pronounced than that of the total removal of patient-derived Gag sequences, as seen in XS clones. In VIS13, reversion of A431V from VIS13BS reduced RC by about half, a reduction that was strikingly lower than that seen with VIS13XS. In VIS18, the reversion of A431V from VIS18BS did not significantly change RC, contrasting with the strong reduction in RC seen in VIS18XS. In VIS16, the change in RC produced by reversion of I437V was again marginal, but in this virus, only a small change in RC was seen when comparing the BS and the XS clone. These results show that NC-SP2 mutations are not sufficient to explain the losses of RC that characterize clones with highly mutated PR and RT sequences and in which patient-derived Gag sequences are absent. They suggest that sequence determinants in NC-SP2-P6 outside of the NC-SP2 cleavage site account in part for this phenomenon, confirming the published results of Myint et al. [19]. Remarkably, however, when NC-SP2 cleavage site mutations were introduced in the XS clones, in which all Gag sequences are derived from NL4-3, we observed almost complete restoration of RC back to levels measured in BS clones. This surprising result shows that in viruses having evolved under prolonged selective pressure by PIs, RC is dependent upon complex interactions between NC-SP2 cleavage site mutations and their environing sequences, while single Gag mutations have a much stronger effect on RC when introduced into an otherwise wild-type background.

### Effect of NC-SP2 cleavage site mutation on Gag processing during viral maturation

Kinetic analyses of HIV-1 Gag cleavage by wild-type PR have revealed that NC-SP2 is one of two sites, together with CA-SP1, where cleavage occurs with slowest kinetics. The importance of NC-SP2 cleavage in infectivity is unclear and has been recently challenged by results showing that mutations obliterating cleavage at this site had little effect on HIV-1 infectivity [21]. Given the remarkable effect of NC-SP2 cleavage site mutations on the RC and on the levels of resistance expressed by viruses with highly resistant PR, we examined extent that these phenotypic changes were reflected by differences in PR cleavage at this particular site. Virions from clones VIS13BS, VIS13BS431A, VIS13XS, and VIS13XS431V were produced by HeLa cells in the absence or in the presence of lopinavir at two concentrations. The protein content of purified virions was analyzed by quantitative western blotting using antibodies against CA and NC.

**Figure 7:**
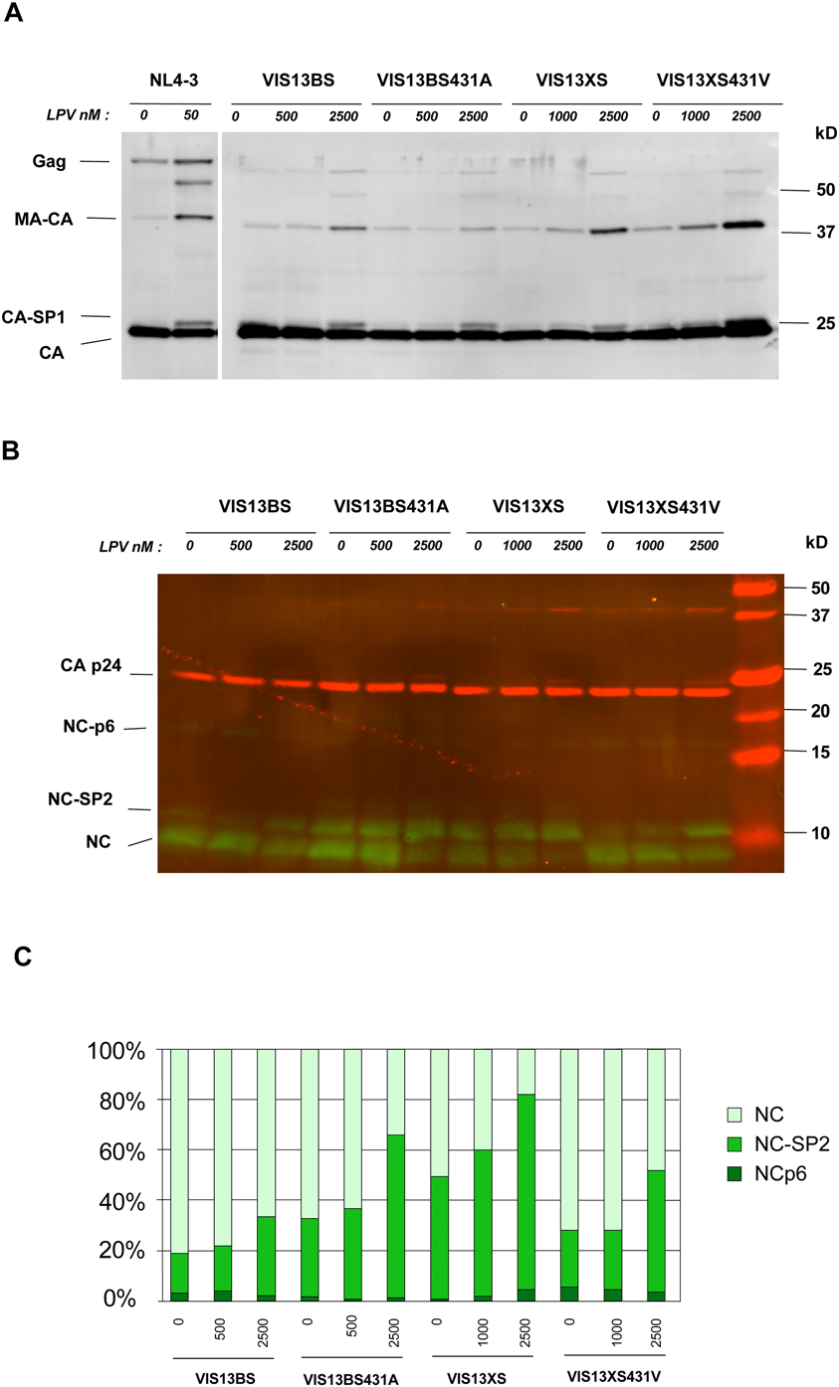
Western-blot analysis of CA and NC products in purified virions produced by viral clones carrying mutations or reversions in Gag cleavage sites. Virions produced by the indicated VIS13-derived clones were purified, lysed, and analyzed by Western blotting using a primary antibody mixture of rabbit anti-CA and goat anti-NC antibodies and secondary antibodies yielding green fluorescence for NC and red fluorescence for CA. Blots were scanned using the Licor Odyssey Infrared Imaging System and quantified with the Licor software. (A) Shows analysis of CA-reactive products on a regular 12.5% polyacrylamide-SDS-gel, while (B) shows NC- and CA-reactive products on a tricine gel yielding better resolution in the low molecular mass range. Quantification of the NC-reactive signals is presented in (C).

Figure 7 shows representative results from one out of three independent experiments. Analysis of the CA-reactive products revealed a strong inhibition of wild-type HIV-1 NL4-3 Gag processing at 50 nM LPV, while processing of VIS13BS derived virus was much less inhibited even at a concentration of 2,5 µM. No significant difference in CA processing was observed when VIS13XS or the derivatives with alterations in position 431 were compared (Figure 7A). In contrast, processing of the NC-SP2 cleavage site was strongly dependent on the amino acid in position 431 (Figure 7B). This was reflected by the relative amounts of fully processed NC and the intermediate cleavage product NC-SP2, while only very small amounts of the NC-SP2-p6 product were detectable in all cases. For virus VIS13BS, which carried all patient-derived Gag sequences including mutation A431V, fully processed NC amounted to ∼80% of all NC-reactive products in the presence of 0,5 µM LPV (Figure 7C). Furthermore, mature NC was still present at >60% of all NC-reactive species in the presence of 2,5 µM LPV, a concentration amounting to approximately twice the IC50 of VIS13BS. Reversion of A431V in VIS13BS (clone VISBS431A) produced a clear change in this pattern with NC-SP2 amounting to ∼65% of all NC-reactive species at 2,5 µM LPV (Figure 7C). A very similar pattern was seen with VIS13XS, in which all Gag sequences were derived from NL4-3. In this case, NC-SP2 amounted to >80% of all NC-reactive species at 2,5 µM LPV and 60% at 0,5 µM LPV. Introducing the A431V mutation into this background reverted the NC/NC-SP2 ratio yielding a NC processing profile comparable to that of VIS13BS at all LPV concentrations.

## Discussion

In this study, we addressed the contribution of Gag to the selection of HIV-1 resistance to PIs *in vivo*. Our results reveal that mutations in the NC-SP2-p6 region of Gag directly contribute to PI resistance in addition to their compensatory role, an effect that correlates with the extent of proteolytic processing of the NC-SP2 cleavage site. In contrast, in these samples from patients having failed multiple lines of antiretroviral therapy, the upstream MA-CA-SP1 region of Gag does not appear to influence PI resistance. These results suggest a new paradigm of resistance evolution *in vivo* that is further supported by recent results from *in vitro* selection experiments [13].

First, we investigated the extent that cleavage site mutations in Gag can promote resistance *per se*, together with their ability to partly compensate for losses of RC that result from mutations in PR. Coevolution of HIV PR and its substrates in PI resistance has been well established for specific mutations in the NC-SP2-P6 region of Gag [7]–[9],[12],[16],[22]. Most studies have emphasized the important role of these mutations in partial compensation of the loss of HIV-1 RC that often results from PR resistance [8],[9],[12],[14],[15]. The mechanism of this compensation has been documented for mutation A431V, which emerges mostly in viruses carrying resistance mutation V82A in PR. Interestingly, this Gag mutation does not act through direct steric compensation of the structural change in the substrate-binding domain of PR, but creates an additional point of contact between the substrate and the substrate-binding cavity of the enzyme. This contact is independent of the presence of resistance mutations, thereby producing a non-specific increase in enzymatic activity at the mutated site [23].

Comparison of recombinant viruses carrying whole patient-derived Gag sequences together with their cognate PR and RT sequences (BS viruses) with recombinant viruses carrying patient-derived PR and RT only (XS viruses) revealed a remarkable contribution of Gag cleavage site mutations A431V and I437V in the NC-SP2-p6 region to the emergence of PI resistance *in vivo* (Table 1). When the cleavage site mutations were removed by site-directed mutagenesis in clones carrying whole patient-derived Gag-PR-RT sequences, 2 to 5-fold reductions in resistance were observed, at least for certain drugs. Furthermore, inserting these mutations into viruses carrying patient-derived PR-RT sequences, but NL4-3-derived Gag sequences usually resulted in viruses whose resistance was equivalent to, or even higher, than that observed for viruses carrying only patient-derived Gag-Pol sequences. The observation that cleavage site mutations A431V and I437V were actually acting as resistance mutations in these highly evolved, highly resistant clinical viruses, is complementary to a recent study [13], which reported *in vitro* selection for resistance to an experimental PI using a laboratory strain of HIV-1 leading to emergence of mutations in SP2 at position 436 and 437 before any changes in PR.

In contrast, the impact of the cleavage site mutations A431V or I437V on viral RC was more nuanced. When cleavage site mutations were removed by site-directed mutagenesis in clones carrying patient-derived Gag-PR-RT sequences, RC was impaired in some cases, but not to the extent that resistance was impaired. Furthermore, a considerably greater loss of RC was observed when the entire patient-derived Gag was replaced by Gag sequences from the reference NL4-3 virus. Thus, although the introduction of cleavage site mutations into viruses carrying NL4-3 Gag sequences and patient-derived PR-RT sequences fully restored viral RC in all cases, the reversion of the same cleavage site mutations from a patient-derived Gag sequence had, at most, only a small impact on RC. Taken together, these findings suggest that other, as yet undefined, mutations had occurred in gag in these patient-derived viruses that partially (VIS 13, VIS16), or completely (VIS18) compensated for the loss of fitness resulting from protease resistance mutations, at least in the absence of PI treatment. These findings are consistent with those of Myint et al. [19], who showed that changes in the p6 region of Gag could participate in partial correction of resistance-associated loss of RC. Furthermore, the context-dependent effects of NC-SP2 cleavage site mutations on RC indicate that these changes can be dually selected: (i) as compensatory mutations restoring RC of PR mutated viruses without further changes in gag and (ii) as direct PI resistance mutations. Upon prolonged treatment, further mutations in Gag outside the cleavage site may render viral RC independent of the A431V or I437V mutations while the effect of these mutations on resistance and thus their selective power *in vivo* persists. The nature of the Gag mutations outside of cleavage site that appear to contribute to viral RC was not investigated in this study. As seen on Figure 2C, numerous polymorphisms, including insertions and duplications in the p6 region of Gag, differed among the viral clones studied here, most of which being frequently observed in HIV-1 sequence databases. Of note, our clones did not carry the polymorphisms described by Myint et al. as being able to exert a compensatory effect on HIV-1 RC. We therefore hypothesize that the RC effect seen in some of our clones following reversion of cleavage site mutations is likely mediated by complex combinations of non-unique natural polymorphisms, which may vary from one virus to another according to their overall Gag genetic context.

The mechanism through which mutations in PR substrates can promote resistance by themselves, independently of their effect on RC, either in the presence or in the absence of resistance mutations in PR has yet to be explained. As discussed earlier, viral resistance to PIs involves a balance between two competing PR ligands: the inhibitor and the Gag or Gag-Pol polyproteins. *In vitro*, the affinity constant of PIs for their target is most often in the sub-nM range, while the affinity constant of Gag or Pol cleavage sites for PR, which varies according to the cleavage site, has been evaluated as being in the mM range [24]. How an increase in the affinity constants of the latter ligand can displace binding of the former is unclear. One explanation, however, may relate to the different stoichiometry of PR relative to its natural substrates at the site of HIV virion maturation [25], compared to that of PR relative to free, active, unbound PIs at these sites. Clearly, further work, involving precise evaluation of intracellular concentrations of PIs at the site of viral assembly, would be needed in order to better understand these phenomena.

The other main observation of our study is that changes in Gag that promote increases in resistance and in RC in highly evolved, highly resistant, viruses are essentially restricted to the NC-SP2-p6 region of Gag. In our panel of viruses, little effect of MA-CA-SP1 regions was measured either in resistance or in RC. The notable interstrain variability in some domains of HIV-1 MA and CA, and in particular the strong variability in the CA-SP1 cleavage site, do not appear to play a significant role in resistance. Two viruses constituted possible exceptions. In virus VIS09, the RC of the AS clone, carrying only patient-derived NC-SP2-p6 in combination with cognate PR and RT patient sequences, was significantly different from that of the BS clone, which carried whole Gag sequences from patient plasma. The resistance levels of the VIS09 AS clone, however, were not significantly different from those measured with the BS clone. In virus VIS20, the same phenomenon was observed, albeit on a smaller scale, and the difference in RC between AS and BS clones did not reach statistical significance. These observations further support the idea that mutations other than NC-SP2-p6 cleavage site mutations can help compensate for losses of viral RC associated with PI resistance, and further suggest that mutations occurring outside of the NC-SP2-p6 region can participate in this process. These mutations do not, however, appear to have appreciable impact on drug resistance.

The absence of effect of MA-CA-SP1 sequences on drug resistance, and their only modest impact on viral fitness, strongly suggests that cleavage events in the NC-SP2-p6 region have a critical impact on resistance and fitness, and may constitute rate-limiting events for HIV virion maturation both in the absence and in the presence of PIs. This conclusion was confirmed by western blot experiments examining the extent of cleavage of Gag proteins in a series of VIS13-derived recombinant and mutants viruses. We first observed that increasing LPV concentration had a similar effect regarding CA processing in all viruses carrying resistance mutations in PR. This was different from the recent *in vitro* selection experiments, where mutations in SP2 at position 436 and 437 increased overall Gag processing [13]. We found, however, that adding or removing the NC-SP2 cleavage site mutation A431V in different VIS13-derived clones did have an effect on the amount of fully processed NC relative to its partially cleaved precursor NC-SP2. This effect was seen both in the absence or in the presence of inhibitor, consistent with the expectations from the structural analysis of the A431V mutant peptide with wild-type and mutant PR [23]. Importantly, the NC/NC-SP2 processing efficiency fully correlated with the resistance phenotypes of the respective viruses. This finding is in apparent contradiction with the results of mutagenesis experiments reporting that mutant virions lacking cleavage at the NC-SP2 site were close to being fully infectious [21]. Several explanations may be proposed to reconcile our findings with those of Coren et al. First, the assay systems used to monitor infectivity are not fully comparable and may differ in their sensitivity to small changes in viral infectivity. Second, these authors have essentially evaluated the effect of mutagenesis of NC-SP2 with wild-type HIV PR from a laboratory molecular clone in absence of protease inhibitor. It can be hypothesized that the rate-limiting nature of cleavage at this site may be more important in the context of primary PR sequences, of resistant PR enzymes, and/or in virions assembled and matured in the presence of protease inhibitors. In this regard, it is noteworthy that the NC-SP2 cleavage site sequence is highly conserved among HIV-1 strains that have never been exposed to PIs, and that in vitro, mature NC has been found to exert optimal chaperone function when compared to other Gag cleavage products, including NC-SP2 [26]. In our experiments, the dramatic effects of mutation A431V on resistance, its context-dependent impact on RC, and its strong influence on the ratio of NC to NC-SP2, strongly argues in favor of a critical role of this cleavage event *in vivo*, in viruses having escaped to pharmacological pressure by protease inhibitors.

## Materials and Methods

### Cells

HeLa cells and P4 cells (HeLa-CD4 LTR-LacZ) were cultivated in Dulbecco's modified Eagle's medium supplemented with 10% fetal calf serum and antibiotics. P4 cells were cultured in the presence of geneticin (500 µg/ml). P4 cells were used as indicator target cells for HIV-1 infection both in resistance and in viral replicative capacity assays [3], [8], [27]–[29]. Infection of P4 cells by HIV-1 virions was monitored in a single cycle of infection, based on the expression of ß-galactosidase, which, in this cell line, is strictly dependent upon induction by the HIV-1 Tat transactivator protein.

### Construction of recombinant viruses with patient-derived Gag, PR, and RT sequences

HIV-1 Gag-Pol plasma sequences from 6 patients with multiple drug resistance were PCR-amplified and cloned into pNL4-3. The patients were recruited from the ANRS 109 “Vista” study, a pilot study aimed at evaluating the virological and immunological consequences of a treatment simplification in patients with multiple antiretroviral drug resistance and no options for suppressive therapy [30]. All 6 patients had a history of failure of multiple lines of combination antiretroviral therapy, which included several protease inhibitors. The recombinant viruses carried different segments of Gag, and/or PR-RT sequences from plasma virus (up to RNAse-H junction) in an NL4-3 background (Figure 3). The GenBank accession numbers for the sequences are VIS09: bankit1176725 FJ649603; VIS13: bankit1176730 FJ649604; VIS15: bankit1176735 FJ649605; VIS16: bankit1176737 FJ649606; VIS18: bankit1176743 FJ649607; VIS20: bankit1176745 FJ649608. RNA was isolated using a Qiagen extraction kit, and the whole-gag protease and reverse transcriptase encoding regions were reverse transcribed and amplified by nested PCR. Nested PCR primers were designed to contain one enzyme restriction site each : BssHII+ (5′ TGC TGA AGC GCG CAC GGC AAG A) and SnaBI- (5′ CCC ATC TAC GTA GAA AGT TTC TGC). The PCR product was digested by BssHII and SnaBI and used to replace the corresponding fragment of pNL4-3, generating a full-length clone (BS clone) carrying the complete gag-protease-RT sequences derived from each patient (between base pairs 711 and 3870 of pNL4-3).

As shown on Figure 3, these 6 BS clones were then used as the source of the gag-pol gene for the construction of three different types of recombinant viruses : i) AS clones, which contained patient-derived NC/p1/p6 protease and reverse transcriptase sequences between the ApaI and SnaBI restriction sites; ii) XS clones, which contained patient-derived PR and RT sequences between the XbaI site and the SnaBI site in association with NL4-3-derived Gag; iii) BX clones, carrying patient-derived Gag between BssHII and XbaI, in association with NL4-3 PR and RT sequences. Introduction of patient-derived sequences into pNL4-3 using the indicated restriction enzymes was made possible by PCR amplification of the corresponding segments of viral genome with primers displaying unique restriction sites. Primers ApaI+ (5′GAA ATT GTA GGG CCC CTA GGA AAA AG) and SnaBI- were used to introduce the restriction sites ApaI at the 5′ and and SnaBI at the 3′ end of the 1862 bp AS fragment. By the same process, XbaI (5′ GGA GCC TCT AGA CAA GGA ACT GTA TCC T) and SnaBI restriction sites were added to the 5′ and 3′ ends of the PR-RT fragment and BssHII and XbaI restriction sites were added to the 5′ and 3′ ends of the Gag fragment.

Six other recombinant viruses were constructed by site-directed mutagenesis. First, to remove and replace the cleavage site mutations (A431V, I437V) of the patient-derived Gag-PR-RT fragment with the wild type pNL4-3 amino acid pattern and second, to add these mutations in the recombinant viruses containing the patient-derived PR-RT fragment. The oligonucleotides used for these mutagenesis experiments are listed in Table S1.

### Protease inhibitor susceptibility assay

HeLa cells cultivated in 25 cm^2^ flasks were transfected with 5 µg of HIV plasmid DNA by the calcium phosphate precipitate method. After 18 hours of incubation, transfected cells were subcultured in triplicate in 96 well plates in the presence of serial dilutions of 6 protease inhibitors. After 30 hours, viral supernatants containing 1–2 ng of p24 antigen were used to infect subconfluent P4 cells in 96 well plates in the presence of DEAE-dextran (15 µg/ml). Forty hours later the single cycle virus titer was determined by quantification of the ß-galactosidase activity in the P4 lysates, using a colorimetric assay based on the cleavage of chlorophenol red-ß-D-galactopyranoside (CPRG). Optical densities in the reaction wells were read at 570 nm with a reference filter set at 690 nm.

### Replicative capacity assay

293T cells cultivated in 25 cm^2^ flasks were transfected with 3 µg of HIV plasmid DNA by the calcium phosphate precipitation method. After 18 hours, the precipitate was removed by gentle washing and fresh medium was added. After a further 24 hours of culture, the supernatant was clarified by centrifugation at 500× g for 15 min and quantified for HIV-1 p24 content by Elisa. P4 indicator cells were infected with two-fold dilutions of the supernatant, representing a range of p24 concentrations from 0.78 ng/ml to 50 ng/ml, in the presence of DEAE-dextran (15 µg/ml). Forty hours later, ß-galactosidase activity in P4 cells was measured by CPRG assay as described above, optical density values plotted as a function of the p24 content in the viral inoculum, and the slope determined by linear regression. Replication capacity was then calculated as the ratio between the slope measured for the tested virus to that of reference virus NL4-3 and expressed as a percentage.

### Immunoblot analyses

293T cells were transfected using the calcium phosphate method in 6-well plates using 2 µg DNA per well. Medium was changed 6 h after transfection and cells and particles were harvested 48 h after transfection. Particles were recovered from clarified culture medium by centrifugation for 1 h at 44,000 rpm through a 20% sucrose cushion in a TLA45 rotor. Cell and particle lysates were separated either on 12.5% standard polyacrylamide gels or on 14% acrylamide tricine gels for superior resolution of smaller proteins as described [31]. Proteins were transferred to Immobilon FL (Millipore) and reacted with a mixture of polyclonal rabbit anti-CA (1∶5,000) and goat anti-NC (1∶3,000; kind gift of Dr. J. Lifson, NCI) followed by a mixture of donkey anti-rabbit IRdye700CW (Licor; 1∶20,000) and donkey anti goat IRdye800CW (Licor; 1∶20,000). Blots were scanned using the Licor Odyssey Infrared Imaging System and quantified with the Licor software as specified by the manufacturer.

### Statistical analyses

IC50 values and fold-changes in IC50 were compared using a one-way ANOVA test. A p<0.05 was considered to be statistically significant. When groups differed significantly, a Bonferroni multiple comparison post-test was performed to make two by two comparisons.

## Supporting Information

Table S1 Primers used for site-directed mutagenesis
(0.03 MB DOC)
