# Supplementary material for: Gag Mutations Strongly Contribute to HIV-1 Resistance to Protease Inhibitors in Highly Drug-Experienced Patients besides Compensating for Fitness Loss
Source: PLoS Pathog. 2009 Mar 20;5(3):e1000345. doi: 10.1371/journal.ppat.1000345 (PMC2652074; doi:10.1371/journal.ppat.1000345)
Supplement: Table S1 — Primers used for site-directed mutagenesis (0.03 MB DOC) [file ppat.1000345.s001.doc]

## Table SI. Primers used for site-directed mutagenesis

| **Primer** | **Restriction Site Inserted** | **Sequence** | **Base Pairs** | Tm °C |
| --- | --- | --- | --- | --- |
| V13BS 431AF | Avr II | 5' CTGAGAGACAGGCTAATTTTTTAGGG 3' | 26 | 55 |
| V13BS 431AR | Avr II | 5' CCCTAAAAAATTAGCCTGTCTCTCAG 3' | 26 | 55 |
| V16BS 437 IF | Bgl II | 5' GGCTAATTTTTTAGGGAAGATCTGGCCTTCCCCC 3' | 34 | 75 |
| V16BS 437 IR | Bgl II | 5' GGGGGAAGGCCAGATCTTCCCTAAAAAATTAGCC 3' | 34 | 75 |
| V18BS 431 AF | Avr II | 5' TGTACTGAGAGACAGGCTAATTTTTTAGGG3' | 30 | 59 |
| V18BS 431 AR | Avr II | 5' CCCTAAAAAATTAGCCTGTCTCTCAGTACA3' | 30 | 59 |
| XS 431V F | Xmn I | 5' CCAGATCTTCCCTAAAAAATTAACCTGTCTCTC 3' | 33 | 76 |
| XS 431V R | Xmn I | 5' GAGAGACAGGTTAATTTTTTAGGGAAGATCTGG 3' | 33 | 76 |
| V16XS 437 VF | - | 5' TTTTAGGGAAGGTCTGGCCTTCCCACAAGG 3' | 30 | 67 |
| V16XS 437 VR | - | 5' CCTTGTGGGAAGGCCAGACCTTCCCTAAAA 3' | 30 | 67 |
